# Implications of Cellular Mechanical Memory in Bioengineering

**DOI:** 10.1021/acsbiomaterials.3c01007

**Published:** 2023-10-05

**Authors:** Oksana Y. Dudaryeva, Stéphane Bernhard, Mark W. Tibbitt, Céline Labouesse

**Affiliations:** † Macromolecular Engineering Laboratory, Department of Mechanical and Process Engineering, ETH Zurich, Zurich 8092, Switzerland; ‡ Department of Orthopedics, University Medical Center Utrecht, Utrecht 3584, Netherlands

**Keywords:** mechanical memory, mechanotransduction, substrate stiffness, cell plasticity, cell expansion, tissue engineering

## Abstract

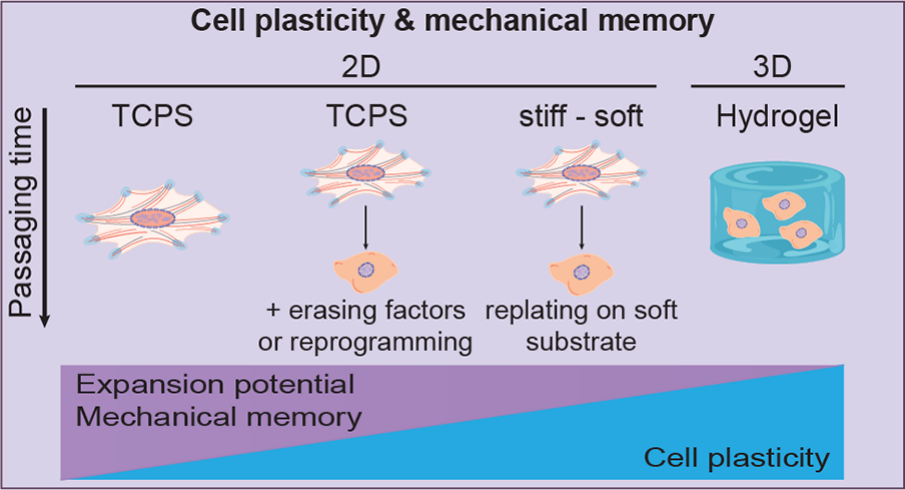

The ability to maintain and differentiate cells in vitro is critical to many advances in the field of bioengineering. However, on traditional, stiff (*E* ≈ GPa) culture substrates, cells are subjected to sustained mechanical stress that can lead to phenotypic changes. Such changes may remain even after transferring the cells to another scaffold or engrafting them in vivo and bias the outcomes of the biological investigation or clinical treatment. This persistence—or mechanical memory—was initially observed for sustained myofibroblast activation of pulmonary fibroblasts after culturing them on stiff (*E* ≈ 100 kPa) substrates. Aspects of mechanical memory have now been described in many in vitro contexts. In this Review, we discuss the stiffness-induced effectors of mechanical memory: structural changes in the cytoskeleton and activity of transcription factors and epigenetic modifiers. We then focus on how mechanical memory impacts cell expansion and tissue regeneration outcomes in bioengineering applications relying on prolonged 2D plastic culture, such as stem cell therapies and disease models. We propose that alternatives to traditional cell culture substrates can be used to mitigate or erase mechanical memory and improve the efficiency of downstream cell-based bioengineering applications.

## 1 Introduction

Many bioengineering applications are based on our ability to culture, engineer, and assemble cells, organoids, and tissues in the lab. Engineered cellular systems are used for fundamental research on biological and pathological mechanisms or reimplanted as therapeutics. Regenerative medicine relies on the ability to extract stem cells from a donor tissue and to grow them outside of the body while maintaining their differential potential prior to their use in vivo. For these applications, it is important that the in vitro culture context (material and duration) does not affect cell phenotype. In vitro cell culture is predominantly based on standard 2D tissue culture dishes or flasks, which fail to replicate the physiological and mechanical properties of the cell niches. A growing body of evidence demonstrates that sustained cell–substrate interactions on these artificially stiff (*E* ≈ GPa) substrates do induce phenotypic changes.

Mechanical forces are key regulators of cell communication, signaling, and gene regulation and implicated in development, organ formation, and cell and tissue function.^1^ Stiffness sensing at cell–matrix adhesions initiates downstream intracellular signaling via mechanotransduction, which varies with the properties of the local environment.^2,3^ In general, cells balance the forces exerted on their surroundings to reach a physical equilibrium with their microenvironment—a phenomenon termed tensional homeostasis—allowing them to respond and adapt to small changes in mechanical stress.^4^ In some instances, the cellular response is transient and the cell quickly re-establishes homeostasis. But, a growing body of evidence suggests that in general the duration (transient or sustained) as well as the rate and timing of mechanical stimuli are important parameters in the regulation of downstream signaling. For example, stiffness-induced expression of transcription factors or of contractility-stimulating factors remains high for several days after cells are replated on softer substrates, as if cells acquire a memory of past environments.^5−7^ This phenomenon of persistent changes in cellular phenotype after a sustained mechanical stimulation is termed mechanical memory.^8,9^ Observations in vitro across multiple cell types support the idea that cells respond to mechanical stress on short but also on long time scales. Together, these studies point to intracellular mechanisms that store or accumulate mechanosensitive factors that modulate cellular plasticity (the ability to adapt phenotype to a given environment).

**Figure 1:**
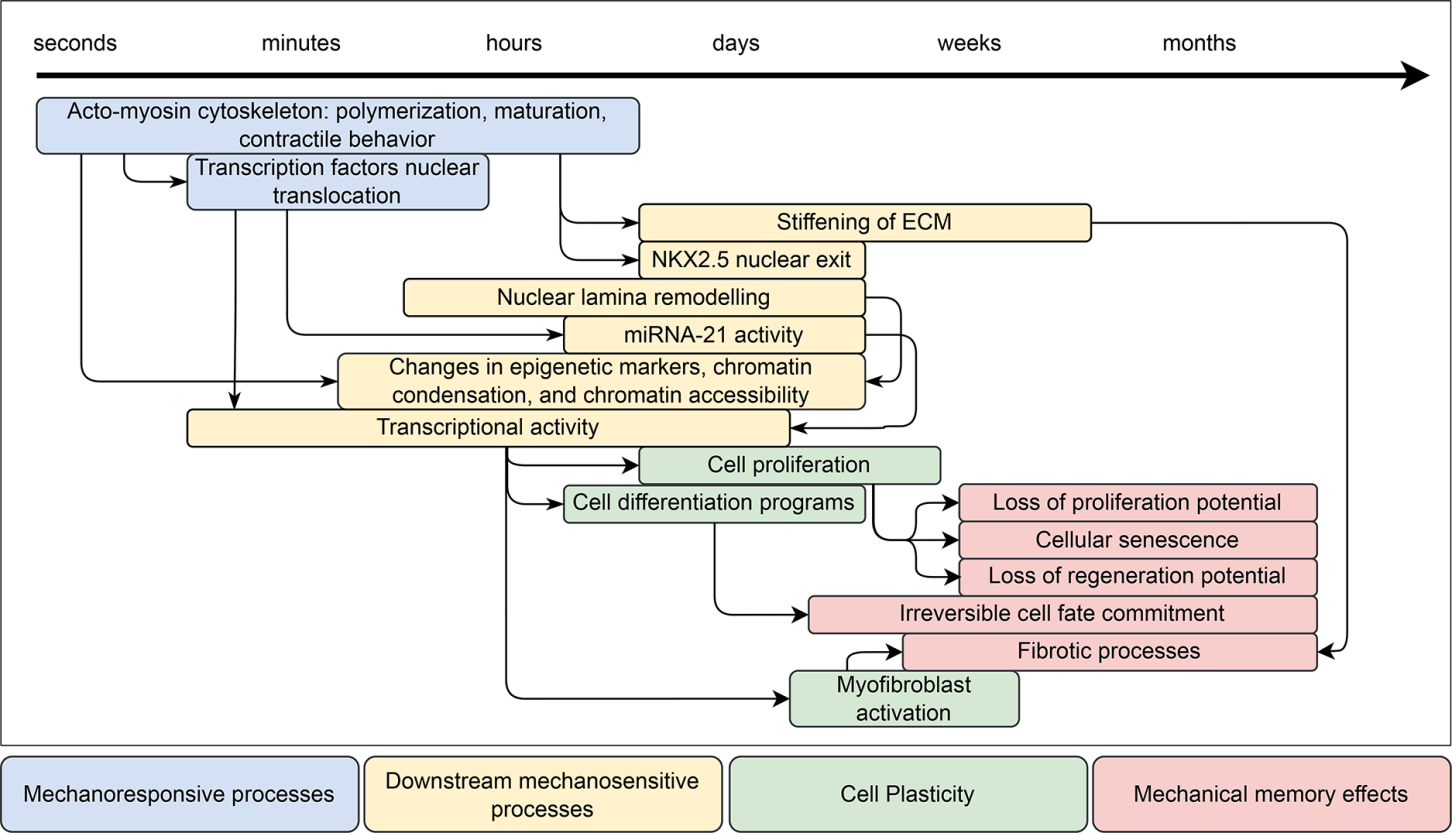
Time scales of processes involved in mechanical memory. Overview of the processes involved at different stages of mechanical activation, mechanotransduction, and mechanical memory. The arrows indicate downstream effects. The color code indicates types of processes: mechanoresponsive processes in blue (directly induced by mechanical stress), downstream mechanosensitive processes that are reversible under transient mechanical stress in yellow, cell plasticity events or example of directed phenotypic changes in green, and mechanical memory effects that emerge (partly) as consequence of sustained mechanical stress in pink.

The intracellular mechanosensitive mechanisms mentioned above are active at different time scales and length scales (nuclear, cellular, extracellular).^10^ Mechanical memory appears to emerge from interactions between cellular processes that feedback onto each other (mechanotransduction, cytoskeleton activity, as well as epigenetic and transcriptional activity) and their relative stability and degradation rates. The sensitivity of these feedback loops is cell-type specific and determines cellular plasticity, such as stiffness-dependent differentiation. How these various processes interact over time is important in determining long-term effects of mechanical stress. To provide context for the timing of events implicated in mechanical memory, we outline approximate time scales of the mechanotransduction and cell plasticity processes discussed in this Review in Figure 1.

Mechanical memory has clear impacts in many bioengineering applications where cells are taken from the body and grown in culture. Prolonged culture on standard labware exposes cells to sustained mechanical stress that affects cell proliferation, differentiation, migration, and metabolic activity.^11−14^ Further, mechanical memory may alter the reparative function of cells following transplantation in the body.^5,15^ Our growing understanding of the bioengineering implications of mechanical memory forces us to consider the context of ex vivo culture systems and encourages the design of alternative culture systems, including soft substrates, 3D encapsulation, or other bioinstructive matrices, that limit unwanted mechanical stress during culture.

In this work, we discuss the role of mechanical memory phenomena in multiple bioengineering applications traditionally reliant on 2D in vitro cell culture. We focus primarily on substrate stiffness as a source of mechanical stress as substrate stiffness has been broadly shown to induce mechanical memory. That said, emerging evidence suggests that other mechanical stresses, including matrix viscoelasticity and fluid viscosity, may also contribute to mechanical memory, and many of the phenomena discussed may translate beyond substrate elasticity alone.^16,17^ We start by discussing evidence for mechanical memory and the potential molecular mechanisms of response to sustained mechanical stress in section 2. In section 3, we highlight how mechanical memory can impact bioengineering applications, from stem cell therapy to disease models, and propose mitigation strategies. We close in section 4 with future implications and challenges for the field.

## 2 Mechanisms of Cellular Plasticity and Mechanical Memory

### Mechanotransduction and Mechanical Memory

Mechanical stimulation impacts cell function through (i) deformation and remodeling of the scaffolding elements (adhesions, cytoskeleton, nucleoskeleton) and (ii) activation of mechanosensitive signaling pathways. This is known as mechanotransduction (reviewed in refs (18−21)). Both transduction paths lead to downstream changes in protein conformation and assembly of protein complexes, modulating their activity and nuclear translocation dynamics. These responses to transient mechanical stimulation (e.g., stiffness) are often reversible, for example mechanosensitive transcription factors may exit the nucleus once cells are transferred from a stiff to a soft substrate. Yet, sustained mechanical stiffness can also induce irreversible functional and phenotypic changes that persist after stimulation has subsided, for example, days after switching cells back to soft substrates. Notable examples include the irreversible activation of myofibroblasts^9^ and the preferential differentiation of mesenchymal stem cells to osteogenic fate.^6,12^ This effect has been termed mechanical memory.^6,8,9,12^ In the following, we specifically discuss the molecular underpinnings of how sustained mechanical stimulation can impact gene expression, cell differentiation, and proliferation states.

Similar to transient mechanotransduction, sustained mechanical stimulation will lead to stabilization and reinforcement of scaffolding elements, deformation of the nucleus, and changes in nuclear import and export of transcription (co)factors and regulatory factors. All of these components (scaffolding elements and mobile factors) can function as effectors of mechanical memory, the turnover rate of which depends on the magnitude and duration of mechanical stress.^22,23^ Seminal studies have identified the cotranscription factor Yes-Associated Protein and paralogous transcriptional coactivator with a PDZ-binding motif (YAP/TAZ) as well as microRNA-21 as such effectors of mechanical memory.^12,15^ Their signaling activity feeds back onto scaffolding elements by promoting reinforcement of cytoskeletal and nucleoskeletal elements and changes in chromatin organization.^24^ Such microstructural changes affect cell phenotype with longer lifetimes than those of mobile factors and are more readily maintained upon transfer to a different environment.

Many effectors of mechanical memory were identified studying the canonical mechanoresponsive process of activation of fibroblast cell types to myofibroblasts. Myofibroblasts are hypercontractile cells that activate in a multitude of tissues upon injury and are essential to tissue repair. They secrete ECM and help close the wound by contracting the tissue. In normal healing, they revert to quiescence or undergo apoptosis after tissue is repaired.^25−28^ In pathological conditions of stiffened ECM and prolonged inflammation, myofibroblasts express characteristics of mechanical memory becoming persistently activated, leading to fibrotic diseases.^29−31^ Sustained mechanical loading and the resulting mechanical memory reinforce the myofibroblast phenotype.^9^ Therefore, we discuss several proposed mechanisms of mechanical memory in light of myofibroblast activation and persistence.

### Adaptation through Cytoskeleton, Cell Contractility, and Nucleoskeleton

**Figure 2:**
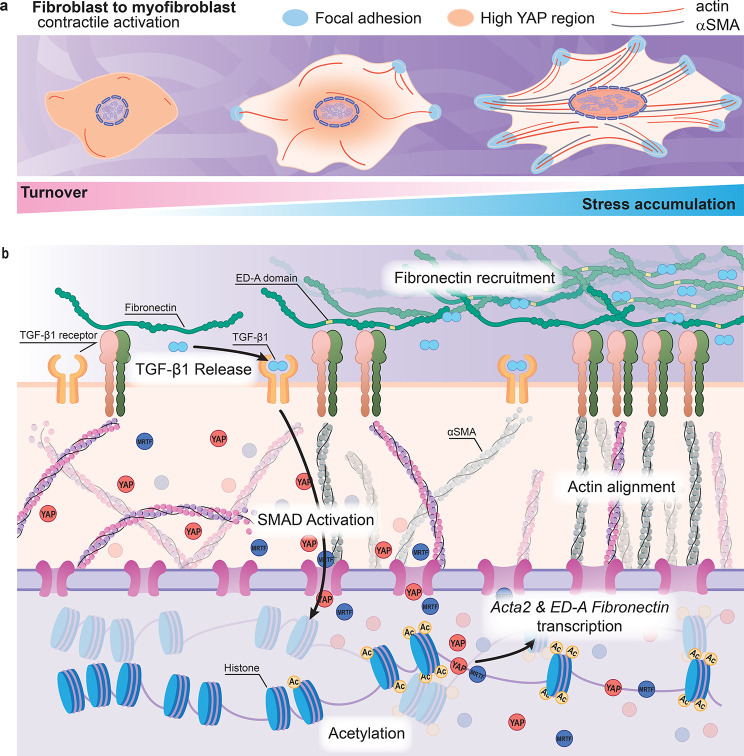
Molecular foundations of mechanical memory. (a) Cells acquire specific phenotypes based on the stiffness of the surrounding matrix. High stiffness leads to higher spread area, increased contractility, and long-lived molecular events. Turnover of cytoskeletal components is higher on soft substrates, whereas stabilization and reinforcement of the cytoskeleton occurs under sustained mechanical stress on stiff substrates. (b) In the ECM, the release of TGF-β1 and incorporation of extradomain A containing (ED-A) fibronectin are hallmarks of fibrotic tissue together with high levels of αSMA (gray) incorporated into contractile stress fibers. TGF-β1 signaling activates the SMAD pathway. Acto-myosin prestress also triggers translocation of YAP and MRTF to the nucleus through stress-dependent opening of nuclear pores. In the nucleus, a drop in HDAC activity increases acetylation and leads to lower chromatin condensation with changes in chromatin accessibility. *Acta2* (coding for αSMA) and *ED-Afibronectin* transcription are activated, while YAP can coactivate gene targets specific to osteogenic differentiation pathways and cell survival.

Persistent stiffness-dependent mechanical alteration of the actin cytoskeleton has been observed in multiple mesenchymal cell types. For example, short-term (approximately hours) memory of strain is held in the cytoskeleton and contractile structures of smooth muscle cells under rapid stretch.^8^ Plastic deformation of the actin cytoskeleton stores mechanical memory of shear stress in astrocytes.^32^ In fibroblasts, prominent stress fibers arise with extended lifetime on stiff substrates and with an increasing orientational order, transitioning from an isotropic to a nematic state.^33^ This ordering becomes more prominent via the formation of microdomains that accumulate and increase in size with substrate stiffness, establishing large-scale order of the actin cytoskeleton. Rheologically, the cytoskeleton transitions from a fluid-like behavior on soft substrates to a solid-like behavior on stiffer substrates. If fibroblasts transition to a myofibroblast phenotype, α-smooth muscle actin (αSMA) is incorporated into stress fibers, rendering them even more stable and more contractile, further contributing to the long-term survival of myofibroblasts even after mechanical stress has subsided and preventing return to quiescence.^34−36^ At advanced stages of maturation, myofibroblasts exhibit features of smooth muscle cells, including microfilaments and dense bodies, evidence of further phenotypic changes.^37^ This cytoskeleton-based feedback loop constitutes an accumulation mechanism by which mechanical stress can create lasting memory in cell architecture and cell state (Figure 2).

The stresses in the actin cytoskeleton in turn lead to structural and mechanical changes to the nucleoskeleton.^23,38,39^ Mechanical stresses are transmitted to the nucleus via anchorage to the linker of the nucleoskeleton and cytoskeleton (LINC) complex, a group of proteins positioned at the nuclear envelope. Together the LINC, nuclear lamina, and associated proteins make up a dynamic nucleoskeleton network that is sensitive to sustained mechanical stress. Lamin A expression and stability not only scales with tissue stiffness and cell contractility^40^ but also with duration of culture.^41^ These structural changes to the nucleus are stable over several days and have direct implications on epigenetic regulation of gene expression. The strain imposed to the nuclear envelope changes the nuclear pore conformation, initiating increased nuclear import rates of mechanical effectors, such as YAP/TAZ.^39^ Prolonged mechanical stimulation leads to an increased retention of YAP^39,42^ and causes nuclei to become persistently larger, less spherical, and stiffer.^43^ In addition, nuclear elongation under high-stiffness conditions affects the spatial arrangement of Lamin A–chromatin interactions, influencing chromatin condensation (discussed in more detail below).

### Maintenance of Transcriptional Regulators

YAP/TAZ comprise an important signaling nexus that influences proliferation, differentiation, and reprogramming by integrating diverse mechanical and biochemical signals, such as ECM stiffness, adhesion ligand density, cell–cell contacts, and the presence of nutrients (reviewed in ref (44)). Mechanosensitive transcription factors localize (nucleus/cytoplasm) upon transient mechanical loading of the cell on time scales of minutes.^45^ The nuclear translocation of the transcriptional coactivators YAP/TAZ is stimulated by actin polymerization and stress fiber formation^46,47^ and regulated by stiffness-dependent nuclear stretching.^39^ Upon sustained mechanical loading, nuclear localization of YAP/TAZ and their corresponding transcriptional activity can persist on the time scale of days^12^ or even become permanent due to positive feedback loops, and as such they serve as effectors of mechanical memory (Figure 2).^12,43^

Myocardin-related transcription factor-A (MRTF-A, also called MKL1/MAL) is another factor sensitive to changes in the ratio of filamentous (F) to globular (G) actin: upon transient mechanical stimulation and actin polymerization, MRTF-A is released by G-actin in the cytoplasm and translocates to the nucleus (Figure 2).^48^ Nuclear MRTF-A acts as a coactivator of serum response factor (SRF),^49^ regulating actin cytoskeletal organization and focal adhesion assembly.^48,50^ This positive feedback loop generates further mechanical stress on the nucleus, enhancing mechanosensitive signaling and thus potentially participating in mechanical memory.^51^ In the nucleus, YAP and MRTF interact through both the SRF pathway and the TGF-β-regulated Smad pathway to govern the expression of αSMA. In the presence of TGF-β signaling that induces activation and nuclear translocation of Smad3, MRTF, and YAP/TAZ exhibit coordinated action in the activation of fibrotic genes. Smad3 along with MRTF and TAZ bind to Smad-binding elements (SBEs) present in the promoter region of the α-SMA gene. Coordinated activities of Smad3, MRTF, and TAZ induce αSMA expression, leading to the phenotypic changes associated with myofibroblast transition.^51^

The positive feedback between YAP/TAZ, MRTF, and reinforcement of the cytoskeleton partially explains how sustained mechanical stress can lead to persistent transcriptional activity, even after the stiffness stimulus is removed. Another mechanism driving persistent αSMA expression is stiffness-dependent nuclear exit of the mechanorepressor NKX2.5. This cardiogenic specification transcription factor has slow nuclear translocation dynamics (several days), and therefore it is only with sustained stiffness stimulus that a change in its activity is observed, leading to increased αSMA expression with increasing passages.^6^ The nuclear exit of NKX2.5 and nuclear import of YAP/TAZ and MRTF upon sustained stiffness both result in persistent activation of αSMA despite acting at different time scales. Their relative activity and turnover times and the translocation dynamics are therefore important in the emergence of myofibroblast phenotype and of long-term mechanical memory.

### Epigenetic Memory: Histone Modifications, DNA Methylation, and microRNAs

Epigenetic regulation is key in the emergence of mechanical memory. Nuclear deformation and more generally persistent actomyosin contractility induce long-lasting epigenetic changes, such as acetylation and methylation of histone tails and methylation of the DNA at promoter regions. These can affect chromatin organization and condensation as well as accessibility to transcription factors.^43,52−56^ Chromatin condensation is regulated by various chromatin remodeling factors, including histone deacetylases (HDACs) whose activity removes acetyl groups on histone tails leading to a local increase in condensation. HDAC nuclear activity is known to be mechanosensitive and has been shown to vary with cell geometry,^57^ cytoskeletal tension,^57^ substrate topography,^58^ substrate stiffness,^43^ mechanocoupling between the nucleus and the cytoskeleton,^59^ and tensile loading.^52^ Cyclic tensile loading of MSCs leads to an increase in chromatin condensation on time scales of minutes.^52,53,60^ Conversely, stiffness-induced mechanical stress appears to initially have the opposite effect, reducing the chromatin condensation within 1 day of culture, through a drop in *HDAC* gene expression.^43^ Importantly, lower chromatin condensation (and therefore greater accessibility) is required to observe phenotypic remodeling, for example activation of fibroblasts to myofibroblasts or their reversion to quiescence.^41^ Prolonged stiffness exposure beyond 5 days as well as high nuclear tension instead drive greater chromatin condensation and prevent reversion to quiescence. In this case, the mechanical memory mediated by the actin cytoskeleton and the tension in the nuclear envelope together drive a persistent myofibroblast phenotype (Figure 2).

DNA methylation on gene promoters is another potential epigenetic effector of mechanical memory. Recent evidence suggests that tissue stiffness could impact DNA methylation. Various fibrotic diseases and cancers demonstrate both abnormally high tissue stiffness and aberrant methylation patterns.^61,62^ Other in vitro investigations demonstrated an acceleration in age-associated methylation changes in cells cultured on stiff 2D plastic compared with in vivo cells.^63^ Together, these studies hint at a possible correlation between sustained mechanical stress, tissue stiffening, and DNA methylation. The link may be YAP, which is known to interact with inhibitors of DNA methylation in gastric cancer cells resulting in stiffness-induced hypomethylation of the YAP promoter and increased oncogenic activation.^64^ Hypomethylation is reversible upon substrate softening, but the extent of reversibility decreases with prolonged mechanical stress. In this manner, YAP and stiffness-dependent epigenetic modifications could induce changes in gene expression via mechanical memory.

Noncoding RNA, such as microRNA (miRNA), also serve as effectors of mechanical memory. The half-life of some miRNAs reaches periods of several days longer than proteins, which usually have turnover rates in hours.^65,66^ Just like transcription factors, miRNAs can be mechanosensitive, respond to transcriptional activation, and regulate αSMA synthesis and cytoskeleton reinforcement. Particularly, miR-19a, -21, -29b, and -200b regulate fibrotic cell programs.^15^ Notably, miR-21 expression increased with stiffness priming (*E* ≈ 100 kPa) and elevated expression persisted up to 2 weeks after transferring cells to soft (*E* ≈ 5 kPa) substrates.^15^ Furthermore, miR-21 knockdown abrogates the effects of mechanical memory induced by stiff priming. The stability of miRNAs makes them more likely effectors of long-term mechanical memory. It should be noted that the production/degradation rate of these factors is also cell-type specific, meaning that the induction and duration of mechanical memory could vary across cell types.^67^

### Extracellular Memory and Mechanical Priming of Cell Migration

While we focus mostly on cellular mechanisms, the extracellular matrix (ECM) is likely also important in mediating the response to sustained mechanical stress in tissues. Most studies investigating mechanical memory use synthetic ECM-mimicking substrates (plastic dishes, elastomers, hydrogels) and cells are replated from stiff to soft substrates or the stiffness is modified in situ. Therefore, the relevance of native ECM in potentiating cellular mechanical memory remains unclear. However, the mechanical properties of the cell microenvironment are known to modulate cell phenotypic changes. For example, stable integrin adhesion to binding motifs, including the Arg-Gly-Asp (RGD) binding motif, plays a role in the maintenance of mechanical memory by stimulating cell contractility.^68^ Diseases, such as fibrosis, in which cell–ECM adhesions increase could thus be more prone to mechanical memory. Fibrosis is accompanied by several processes potentiating cell–ECM adhesion. Lysyl oxidases (LOX) stabilize fibrous ECM proteins, such as elastin and collagen, through oxidation of lysine residues, thereby initiating the formation of covalent cross-linkages and enhancing integrin signaling.^69^ Additionally, myofibroblasts increase expression of extradomain A containing (ED-A) fibronectin, which in turn potentiates the TGF-β1 pathway and αSMA expression (Figure 2). TGF-β1 itself activates alternative ED-A fibronectin splicing that reinforces cell adhesions, fibrotic ECM, and TGF-β1 signaling.^37^ The positive feedback loop between the ECM and TGF-β1 signaling contributes to the persistent fibrotic cell phenotype in mesenchymal cells.

In epithelial cells, ECM deposition provides a stable footprint biasing migratory phenotype (directionality and migration speed),^70^ creating a spatial collective memory of migration trajectories. Mechanical memory in cell migration may be relevant in vivo to the initiation of metastasis, where cancer cells exit a stiff tumor environment to migrate through softer tissue. Recent studies have shown that this memory in oral squamous cell carcinoma cells is mediated by cell contractility and the activation of Akt and FAK pathways.^71^ In addition, tumor-conditioned stromal ECM alters the pro-angiogenic signaling profile of newly seeded cells, meaning that the initial force-dependent ECM remodeling by tumor cells can impact long-term tissue fate.^72^ Similar effects may influence the migration of immune cells from swollen lymph nodes to various tissues. In summary, both the components of the cell-deposited ECM and its conformation constitute a unique fingerprint of past cellular identity and mechanical stress and may serve as an important potentiator of(extracellular) mechanical memory.

Extracellular mechanical memory has been extensively studied in cancer cells and it was proposed more than a decade ago that the ECM could be thought of as a biological memory-storage device with information being written in the cross-linking status of the ECM.^73^ Aberrant ECM stiffening can promote disease progression including tumor growth and metastasis.^74^ High ECM cross-linking and integrin signaling increase breast cancer malignancy.^69^ Stiffness priming (also called mechanical conditioning) has also been shown to promote metastasis of breast cancer through prolonged expression of YAP target RUNX2 and changes in chromatin accessibility.^75^ We note, however, that important factors in mechanosensitive pathways, including integrins, are often dysregulated in cancer, such that some transformed cells exhibit reduced ability to sense substrate stiffness.^76^ In addition, the correlation between cell contractility and cell adhesion can be disrupted in weakly adherent cancer cells.^77^ Thus, in some tumor cells, particularly in metastatic cells, mechanical priming may be less relevant. Altogether, it is possible that mechanical memory participates in initial cancer progression, but this is likely dependent on the type of cancer and the mutational background.

### Time Scales of Mechanical Memory

The evidence thus far on mechanical memory converges on two points. First, memory of past stiffness is dose dependent, increasing with priming time until the effects become irreversible. In many short-term activations, the processes are reversible, and the reversibility of various mechanoactivation mechanisms was recently reviewed in more detail.^78^ The critical time before irreversibility likely depends as much on cell state (e.g., its mechanosensitivity, its differentiation potential, and the cell cycle stage) as on the time-dependent signaling pathways active during specific differentiation events. To our knowledge, no study has systematically tested how mechanical memory depends on the magnitude of stiffness difference (although this has been theoretically explored^79^), but we speculate that this effect would be nonlinear, as the mechanical load on cells from cell–ECM traction forces scales nonlinearly with substrate stiffness.^80^ The second converging point is that there are multiple effectors of mechanical memory whose activity depends on cell types and type of mechanical stress. The characteristic time constants important for mechanical memory are the duration of mechanical priming and the stability of proteins and epigenetic factors as well as the compound response time of cytoskeleton–transcription feedback loops (Figure 1). In recent computational studies, these parameters alone were sufficient for mechanical memory.^67,81^ These time frames should be carefully considered in the choice of cell and tissue culture systems to avoid unwanted memory-related phenotypes.

## 3 Bioengineering Implications of Mechanical Memory

Bioengineering and biomedical applications rely on the ability to culture cells in vitro, allowing cell expansion, differentiation, reprogramming, as well as detailed biological investigations. Standard in vitro culture uses glass Petri dishes or tissue culture polystyrene (TCPS). However, the supraphysiologic stiffness (*E* ≈ GPa) of standard 2D culture affects cell function via mechanotransduction pathways.^82,83^ Further, long-term culture on TCPS induces unintended effects that limit the efficiency of downstream applications of cultured cells.^84,85^ While cell proliferation often positively correlates with stiffness on short time scales,^82^ prolonged culture on stiff substrates decreases proliferation potential^84^ and often induces differentiation and loss of regenerative potential. The emerging evidence of mechanical memory suggests that these effects can be long lasting, can increase with culture time, and are not always reversible (Table 1). Therefore, careful consideration must be given to the duration and context of in vitro culture systems for any downstream application.

**Table 1:** Examples of Observed Mechanical Memory Phenomena, Potential Mechanisms Involved, and Potential Strategies To Mitigate the Effects

cell type	culture system and mechanical stress	observed phenotype	potential mechanism(s)	potential strategies(s)	ref
muscle stem cells	culture on aberrantly stiff (*E* ≈ GPa) or soft (*E* ≈ 1 kPa) substrates	poor engraftment in an in vivo model as compared with native cells	proposed to be linked to changes in cell shape, cytoskeletal arrangement, and cell signaling	culture on substrates that mimic native tissue stiffness (*E* ≈ 12 kPa)	(5)
lung myofibroblasts	prolonged culture on stiff (*E* >100 kPa) elastomer or plastic substrates	persistent myofibroblast activation with αSMA expression	elevated levels and activation of TGF-β1	culture on soft (*E* ≈ 5 kPa) elastomers	(9)
mesenchymal stem cells	extended culture on stiff (*E* ≈ 10 kPa) hydrogels	persistent activation and preferential osteogenic differentiation	sustained YAP/TAZ nuclear localization	substrate softening (to *E* ≈ 2 kPa)	(12)
mesenchymal stem cells	culture on stiff (*E* ≈ 10 kPa) collagen-coated hydrogels	myofibroblast activation and excessive contractility	downregulation of NKX2.5	culture on soft (*E* ≈ 0.3 kPa) collagen-coated hydrogels	(6)
mesenchymal stem cells	culture on stiff (*E* ≈ 100 kPa) fibronectin-coated PDMS substrates	myofibroblast activation	MRTF-A nuclear localization followed by upregulation of miRNA-21	modulation of miR-21 expression	(15)
mesenchymal stem cells	serial passaging on TCPS	loss of differentiation potential, cellular sensescence		serial passaging on soft (*E* ≈ 5 kPa) substrate	(84)
mesenchymal stem cells	serial expansion on TCPS	decrease in proliferation and loss of cell function		expansion on PEG hydrogels (*E* ≈ 1 kPa)	(86)
mesenchymal stem cells	culture on stiff (*E* ≈ 30 kPa) PEG hydrogels	persistent mechanical activation, high chromatin acetylation	increased nuclear YAP translocation and histone acetylation	substrate softening to *E* ≈ 2 kPa	(43)
fibroblasts from the aortic valve	culture on stiff (*E* ≈ 13.5 kPa) RGD-functionalized hydrogels	myofibroblast activation	increased HDAC activity, reduced chromatin accessibility	supplementation of actin-disrupting factors	(41)
epithelial cells	culture on stiff (*E* ≈ 50 kPa) polyacrylamide substrates	increased collective epithelial migration	sustained nuclear translocation of YAP	shRNA-mediated depletion of YAP	(13)
oral squamous cell carcinoma	culture on stiff (*E* ≈ 20 kPa) substrate	faster cell migration	AKT and FAK signaling activation	use of FAK or AKT antagonists	(71)
breast cancer cells	mechanical conditioning on stiff (*E* ≈ 8 kPa) substrates	higher chromatin accessibility, higher metastasis potential	high RUNX2 transcriptional activity	blocking RUNX2 phosphorylation, soft substrate conditioning	(75)
neural stem cell differentiation	culture on stiff (*E* = 72 kPa) hydrogels	suppressed neurogenesis	overexpression of YAP	YAP knockdown by shRNA, ablating YAP−β-catenin interaction	(87)
neural stem cell differentiation	culture on stiff (*E* = 73 kPa) hydrogels	suppressed neurogenesis	upregulated YAP activity	upregulation of pAMOT to inhibit YAP and promote β-catenin	(88)
chondrocytes	prolonged culture on stiff (*E* ≈ 25 kPa) hydrogels	chondrocyte dedifferentiation	decreased occupancy levels of H3K9me3 on dedifferentiation genes	increasing levels of H3K9me3 by inhibiting demethylases of H3K9	(89)

### Mechanical Memory Drives Myofibroblast Activation

Fibroblasts and fibroblast-like cells are among the most mechanosensitive and contractile cells and are prone to fibrotic activation. Mechanical responsiveness of fibroblasts is essential in wound healing and tissue repair, while prolonged mechanical stimulation leads to irreversible myofibroblast activation and fibrotic disease. In bioengineering applications involving fibroblasts, for tissue modeling or repair, we seek to avoid their unwanted mechanical activation towards myofibroblasts. In this regard, the exposure of cultured cells to sustained mechanical stress is an important consideration for the design of culture and delivery systems. This is particularly relevant as stiffness-induced phenotypic changes in fibroblasts persist even after transfer to soft substrates.^9^ Primary fibroblasts isolated from rat lungs were cultured on native-like stiffness conditions (*E* ≈ 5 kPa) and on stiff substrates representative of fibrotic tissues (*E* ≈ 25–100 kPa). After 6 days, myofibroblast activation increased with substrate stiffness.^9^ The myofibroblast phenotype persisted even up to 2 weeks after transfer to softer (*E* ≈ 5 kPa) substrates,^6,15^ demonstrating the long-lasting mechanical memory effects of stiffness priming in fibroblasts. A similar observation was made with valvular interstitial cells (VICs). VICs cultured on soft substrates (*E* ≈ 7 kPa) maintained a quiescent phenotype and were transcriptionally closer to the freshly harvested VICs, whereas even a single passage on TCPS or stiff (*E* ≈ 32 kPa) substrates induced myofibroblast differentiation and overexpression of fibrotic genes.^7^ In situ substrate softening after 3 days deactivated myofibroblasts, partially restoring the quiescent fibroblast population.^90,91^ Similarly, stiffness priming induced a myofibroblast phenotype in rat hepatic stellate cells that was only partially reverted during gradual substrate softening and rapidly reactivated upon subsequent stiffening.^92^ Together, these studies highlight how long-term culture of fibroblasts or fibroblast-like cells on stiff substrates can induce a persistent myofibroblast population. Therefore, any time fibroblasts or fibroblast-like cells are grown in culture it is important to account for direct or latent effects of mechanical memory on myofibroblast activation in the population.

### Stem Cell Therapies: Mechanical Memory Affects Expansion and Engraftment

The development of stem cell therapies, including those using adult human mesenchymal stem cell (hMSCs), has increased in the past years with >1400 ongoing clinical trials, several hundred completed trials, but only a handful of hMSC therapies having been approved.^93,94^ Due to the potential of stem cells to serve as a replacement for a variety of cell types, such as osteoblasts, adipocytes, and chondrocytes, stem cell therapies can be employed for tissue regeneration and as potential treatments for a variety of pathologies, such as cardiac disorders, autoimmune diseases, or even cancer. Stem cell therapies require the delivery of a large number of cells per patient. One therapeutic dose typically requires around 100 million cells injected to an injury site to initiate a repair response.^93^ This quantity of cells is difficult to obtain directly from donors. For example, MSCs are mainly harvested from bone marrow aspirates or adipose vascular fraction, where 0.001–0.01% of cells are suitable for therapy.^86^ Thus, in vitro expansion is needed to generate a therapeutic number of cells. Cell expansion is typically performed on materials with properties that differ from the cell niche.^96^ The challenge lies in the fact that in vitro culture on standard TCPS (*E* ≈ GPa) reduces proliferation and differentiation potential over time compared with freshly harvested or early passage MSCs.^5,7,84,97^ A trade-off must therefore be found between cell expansion and maintenance of stem cell potential.

**Figure 3:**
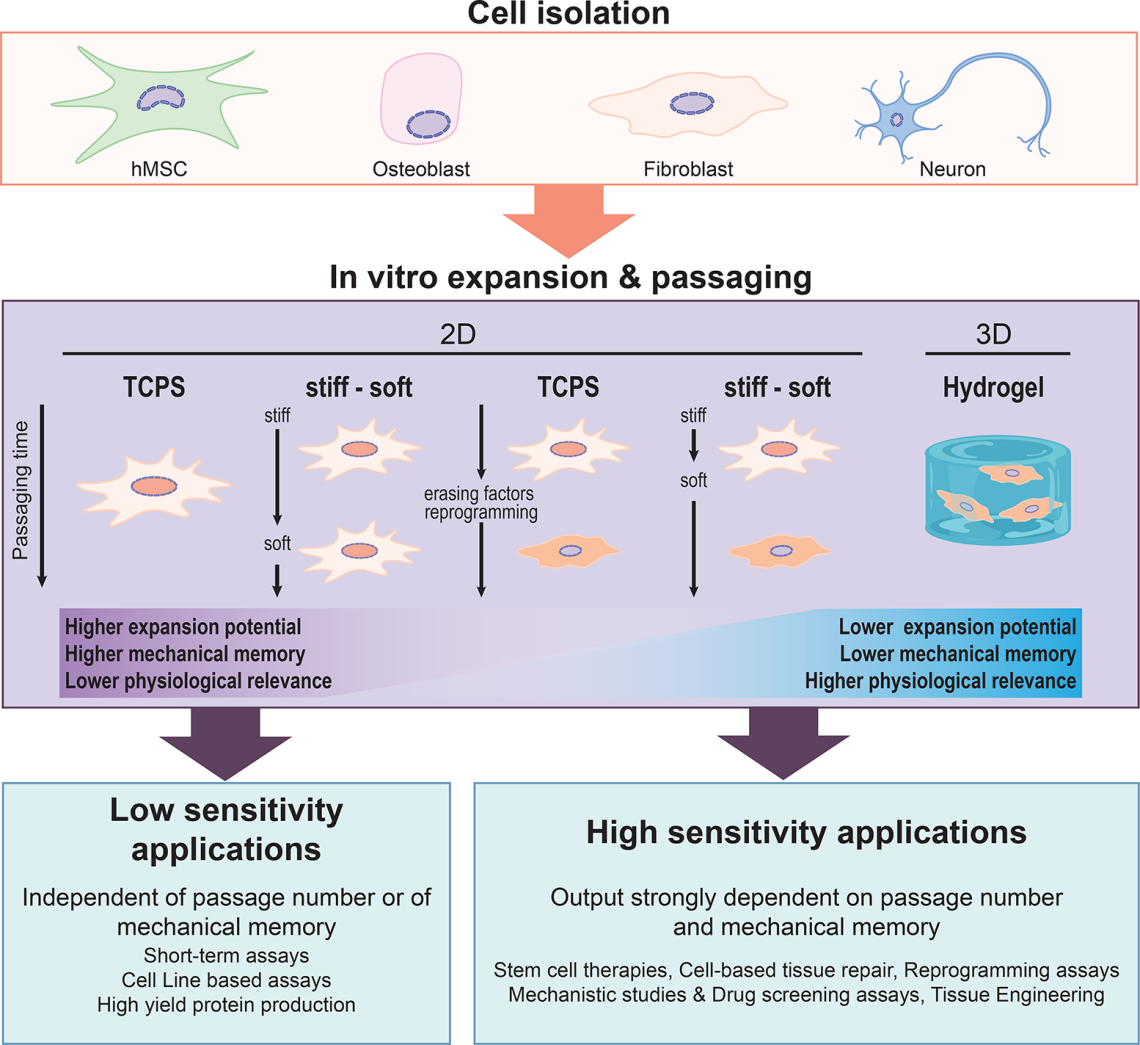
Bioengineering applications that can be affected by mechanical memory can be a factor and strategies to mitigate undesired effects. The level of mechanical memory will depend on the starting cell type and its mechanosensitivity as well as on the final application and duration of passaging. Low-sensitivity applications (cell-based or molecular assays on time scales of approximately hours or <1 passage) should not be affected by mechanical memory. Applications requiring >1 passage of in vitro cell culture are more sensitive to mechanical memory. Options to mitigate the effects are to transfer cells from stiff to soft substrates early in the process to avoid persistent YAP nuclear localization (orange hue). Alternatively, memory-erasing factors or reprogramming strategies can be used together with TCPS culture (see text for details). Finally, 3D cell culture could mitigate the effects of mechanical memory using physiologically relevant systems, but this requires further cell-type-specific studies.

Mechanical memory affects stem cell potential via the stiffness of in vitro expansion substrates and the duration of the expansion. hMSCs grown on TCPS lose their proliferative capacity and multilineage differentiation potential after several passages,^84^ show attenuated expression ofMSC-specific markers, and upon further expansion become senescent.^84,98^ At high passage on TCPS, populations also become biased toward osteogenic differentiation and lose adipogenic differential potential.^12,85,98^ However, hMSCs and adipose-derived stem cells maintain their proliferation and differentiation potential and delay senescence when cultured on soft (*E* ≈ 5 kPa) gels even at high passage (approximately passage 18).^84,85,99^ The time point at which stiffness priming permanently biases cell phenotype appears to be several days. Following short-term exposure to stiff substrates (*E* ≈ 10–15 kPa), VIC quiescence and hMSC adipogenic potential are restored by softening the substrates (*E* ≈ 2–5 kPa).^12,41,43,86^ However, the time window for reversibility was limited to 5–7 days of culture on stiff substrates in both cases. Beyond this time point, expression of preosteogenic transcription factors or of fibrotic markers persists even after substrate softening (Figure 3),^12^ driven by high YAP nuclear localization and changes in chromatin accessibility, as discussed in section 2. Thus, to avoid loss of proliferation and differential potential, short expansion times (<5 days or 2 passages) on TCPS or stiff substrates are favored.

Aside from the proliferation and differentiation potential, successful engraftment of stem cells in tissues is a critical step. Stem cells used in cell therapy have important paracrine signaling function (secreting growth factors and immunoregulatory cytokines). Their signaling activity, which depends on cell state, contributes to tissue regeneration and repair.^100^ Stem cells can be used at lower therapeutic dose for stimulating regeneration compared to direct repair. After in vitro expansion, these stem cells are reimplanted to graft onto the target tissue and need to maintain their regenerative capacity. Muscle stem cell engraftment in vivo depends on the history of culture conditions in vitro.^5^ Freshly harvested muscle stem cells were expanded on hydrogels with stiffness matching the properties of the muscle niche (*E* ≈ 12 kPa) or on TCPS before reimplanting them into injured mice.^5^ The rate of engraftment and regeneration capacity was highest for cells precultured on substrates with the stiffness of muscle tissue. Placing muscle stem cells in a muscle niche in vivo is not sufficient to restore their regeneration potential, instead the substrate stiffness used for cell expansion determines in vivo efficacy (Figure 3). Grafting success of organoids shows a similar dependence to substrate stiffness. Human pluripotent stem cells (hPSC)-derived kidney organoids can be implanted into a chick chorioallantoic membrane (CAM, an extraembryonic tissue providing vascularization). The organoids display increased renal vesicle and nephron structure formation as well as improved differentiation when the cells are differentiated on soft (*E* ≈ 1 kPa) hydrogels with similar stiffness to the CAM prior to implantation.^101^ Cells remember past mechanical environments, and this mechanical memory is preserved even upon engraftment in vivo. Therefore, both for stem cell expansion and for downstream engraftment and tissue repair, stem cells should be expanded on substrates that limit stiffness-induced mechanical stress and preserve their regenerative capacity (Figure 3).

### Disease Models and Mechanistic Investigations

Embryonic stem cells and induced pluripotent stem cells (iPSCs) are frequently used to generate specialized cell types in vitro, to improve disease models, and to identify novel therapies. While many biochemical and reprogramming strategies exist to guide stem cell fate, physical factors also contribute to differentiation.^83,102,103^ Differentiation of adult neural stem cells (aNSC) from iPSCs is stiffness dependent; neurogenesis is more efficient on soft (*E* ≈ 0.3 kPa) substrates compared with stiff (*E* ≈ 3 kPa) substrates.^87,88,104^ Prolonged culture on stiff substrates (beyond 2 days) can irreversibly affect the outcome of differentiation.^87^ For these multipotent stem cells, the first 36 h of differentiation were the most sensitive to stiffness priming.^87^ In this time frame, neurogenesis increased on softer substrates. This window corresponds to the duration of Wnt signaling required for neurogenesis, which is promoted in low-contractility conditions by the sequestering of YAP by Angiomotin (AMOT) but hindered in high-contractility conditions through the inhibitory action of YAP on β-catenin.^87,88^ These observations suggest that mechanical memory may be mediated by both cell contractility and signaling activity of mechanosensitive factors in aNSCs and that culture context is critical in the bioengineering of neural cell therapies.

2D and 3D in vitro models are an important part of the drug development pipeline for both mechanistic and drug screening studies. These studies can be impacted by cellular mechanical memory. Long-term culture on stiff substrates will generate cytoskeletal prestress and change the nuclear conformation, affecting various signaling pathways, transcription, and gene expression, biasing results that rely on these signaling pathways. Interventions targeting cell proliferation, differentiation, migration, as well as metabolic activity or drug uptake rate should be tested in mechanically naïve cells and compared to stiffness-primed cells, since all of these cellular functions are known to be either stiffness sensitive or regulated by YAP activity and thus subject to mechanical memory effects.^14,105,106^ For example, interventions targeted at improving wound healing through improved cell migration can be biased by prior stiffness priming of epithelial cells. Cell migration speed is known to increase with substrate stiffness.^107,108^ Stiffness priming (*E* ≈ 50 kPa) will lead to higher migration speed even 2 days after replating on softer matrices (*E* ≈ 0.5 kPa), maintaining the markers of fast cell migration including high contractility, large focal adhesions, and alignment of actin filaments.^13^ The stiffness and duration of mechanical priming can enhance YAP activation and the duration of mechanical memory.

### Bioengineering Strategies To Mitigate or Erase Mechanical Memory

With the growing understanding of how culture context can influence cell function and fate, it is important to consider how to avoid undesirable effects of mechanical memory in cell-based bioengineering applications. One strategy is to replace TCPS with engineered substrates that better mimic the mechanical properties of the in vivo niche for cell growth and passaging. The use of soft (*E* ≈ 5 kPa) substrates enables longer retention of MSC differentiation potential compared with cells cultured on TCPS.^84^ After 18 passages on soft substrates, no senescent markers were detected in cultured cells and the cell shape remained unchanged. MSCs cultured on soft substrates exhibited higher rates of proliferation, which is critical for clinical applications, though this should be investigated more generally with different cell types and substrate types. In addition, combining soft substrates with adhesion cues improves MSC potential for bone tissue engineering. MSC spheroids grown in RGD-modified alginate hydrogels showed increased survival, higher angiogenic growth factor production, and greater mineralization than spheroids grown in unmodified alginate.^109^ Finally, application of factors that reduce cytoskeletal tension, YAP activation, and TGF-β1 signaling can be explored. For example, the use of cell–cell binding motifs (HAVDI) in addition to cell–ECM binding RGD motifs in engineered cultures may limit mechanical memory in hMSCs.^68^ The use of growth factors, such as fibroblast growth factor 2 (FGF-2), that compete with TGF-β1-mediated signaling antagonized the expression of αSMA and maintained proliferation rates in pericytes.^110^ Further chemical agents, such as verteporfin, attenuated YAP signaling both in vitro and in vivo, limiting fibrotic phenotypes in fibroblasts.^111^

Using bioinstructive soft substrates for in vitro culture can be leveraged at different stages, either partially or completely replacing TCPS, or as a restoring phase after cell expansion.^112^ Partial TCPS culture would consist of single-passage TCPS cell amplification (<5 days of culture) followed by replating on soft substrates for several days. This strategy should allow faster cell expansion than exclusive soft substrate culture. Post-expansion culture on soft hydrogels may potentially also be used after TCPS expansion to erase the mechanical memory. One study successfully restored the multipotency of hMSCs cultured on TCPS by transferring them to soft (*E* ≈ 1 kPa) hydrogels after serial passaging on TCPS.^86^ After transfer to soft hydrogel matrices, hMSCs regained markers indicative of stem cell identity and their capacity to secrete cytokines, important for regenerative medicine applications,^86^ although this was not fully successful in another study using adipose-derived stem cells.^85^ A complementary strategy would be to stimulate stem cell potential through FGF-2-mediated stimulation of the ERK pathway or to prevent telomere shortening with antiaging compounds. This will likely only mitigate but not erase mechanical memory.

Directly targeting mechanical memory factors is the most promising strategy so far to erase memory. Following the identification of miR-21 as a memory factor, RNA-silencing approaches erased the mechanical memory of myofibroblasts to reduce the scarring in a mouse model of wound healing.^15^ In another study, mechanically decoupling the nucleus from the cytoskeleton was found to be more efficient to deactivate myofibroblasts than targeting chromatin acetylation.^41^ Going further, cells can be reprogrammed using tailored substrates.^113^ Fibroblasts were successfully reprogrammed to pluripotent stem cell-like spheroids and then rejuvenated in collagen I matrices to fibroblasts with increased matrix secretion capacity and increased contractility.^113^ In vivo, partial reprogramming with transient expression of Yamanaka factors also shows rejuvenating effects with reversal of age-related epigenetic, metabolic, and transcriptomic changes,^114,115^ although the effect of partial reprogramming on mechanical memory factors remains to be tested. Memory-erasing and reprogramming approaches should be used with caution as most of the current evidence on mechanical memory suggests that the effects are dependent on the stiffness-priming time, and therefore, replating cells on a soft substrate may not always suffice to restore stem cell potency and normalize quiescent function. Further investigations are needed to identify other memory-related factors (epigenetic and transcription factors) and strategies to erase them.

## 4 Outlook

Sustained mechanical stress can introduce irreversible consequences on cell and tissue function. This concept of mechanical memory is established for multiple cell types and different contexts, and a particular emphasis has been placed on mesenchymal stem cells and myofibroblasts, two cell types that are natively present in environments of high mechanical load. The majority of the observations related to mechanical memory in bioengineering are based on effects of stiffness priming, and it is possible that similar phenomena occur with other types of repetitive mechanical stress, such as tensile loading or shear stress. Similarly, surface curvature,^116^ surface nanotopographical cues,^117^ and substrate adhesion strength^118,119^ are all known to activate mechanosensitive pathways that directly or indirectly regulate stem cell differentiation. Whether long-term stimulation on these specific types of substrates reduces cell proliferation or differentiation potential would be interesting to investigate. Independent of the type of mechanical activation, the effects of sustained mechanical stress could be mediated by a range of cellular factors, from the stability of contractile cytoskeleton structures and the activity of transcriptional regulators, up to modifications in chromatin conformation and the extracellular matrix. Of note, the expression and lifetime of diffusing transcriptional regulators, e.g., YAP/TAZ, MRTF-A, and NKX2.5, are also important as they regulate gene expression programs downstream of mechanical stimulation. All of this information highlights the need to better control the in vitro expansion of stem cells to avoid mechanical memory-related loss of cell plasticity and regenerative potential. Cell culture context matters, and it can have deleterious impacts in bioengineering applications, including the therapeutic use of stem cells, the design of microphysiological models, or the study of molecular signaling pathways. The design of culture systems that can maintain cells in a near-physiologic state without activating mechanical memory remains a critical need in the bioengineering community.

We note that the concept of memory in cells is not new, and in particular, epigenetic changes are known to be transmitted through cellular generations, establishing a memory of chromatin accessibility and transcriptional activity. Epigenetic mechanisms may here be involved in the maintenance and transmission of mechanical memory across cell divisions. In addition, many instances of nuclear mechanotransduction have been described in which mechanical forces can impact the organization of the nuclear lamina and chromatin, driving transcriptional changes.^120^ However, current observations of mechanical memory appear to be more than the combination of nuclear mechanotransduction and epigenetic effects as mechanical memory is also held independently of transcriptional activity in the contractile structure of the cytoskeleton and because the time scales and duration (or dose) of mechanical stress differ. We strived to distinguish mechanotransduction (transient stimulation, transient response) from mechanical memory in which the time scale of mechanical stress and transcriptional changes occur over days. However, it remains possible that there is mechanistically no distinction at the molecular scale between these effects, but the appearance of mechanical memory emerges as a consequence of longer observational timeframes. Regardless, we believe that the impacts on bioengineering applications are of importance.

Several questions remain before we fully understand the scope of mechanical memory in bioengineering. First, the reversibility window during stiffness priming varies between a few days to multiple passages depending on assay and cell type; therefore, it is difficult to define precise guidelines for all cells. Second, the ability to erase mechanical memory or reprogram cells to a mechanically naïve state should be more extensively explored. Potential strategies include application of memory-erasing factors (e.g., miR-21) in vivo, use of mechanosuppressing drugs (e.g., YAP inhibitors such as verteporfin), strategies to soften the tissue or ECM, or physical confinement with patterned substrates to force mechanical reprogramming. Epigenetic reprogramming for cell rejuvenation is also promising, but it remains to be confirmed whether this approach is sufficient to erase mechanical stress history. New reprogramming strategies could improve yield during cell expansion without compromising stem cell potential for direct translational benefit. Further investigations are needed to gain a comprehensive understanding of the molecular basis of mechanical memory, confirm the role of putative mechanical memory effectors, and determine how their half-life depends on the niche state and the cell state. It would be useful to measure the turnover/degradation rates of these factors, including YAP/TAZ and miR-21, in the context of in situ stiffness changes and with loss of cell plasticity.

Little is known about mechanical memory in 3D culture and how the duration of stiffness priming and thresholds impact cell states in 3D. The role of stiffness is less straightforward in 3D due to confounding effects of confinement within stiff matrices. Cells do not spread as much and are less contractile when confined in dense matrices, and matrix degradability and/or porosity are necessary to allow cell spreading.^121−123^ Nonetheless, preliminary results from 3D studies parallel those from 2D. In 3D microniches, HDAC3 activity inversely correlated with cytoskeleton engagement and YAP nuclear activity,^122^ mirroring previous findings in 2D.^43,57,59^ Differentiation outcomes also follow previous 2D findings with higher relative osteogenesis compared to adipogenesis in smaller niches that activated cytoskeleton and myosin contractility. In 3D culture of neural tube organoids, mechanical stimulation had an irreversible effect on floor plate differentiation patterns.^101,124^ In noninvasive cancer cells, mechanical priming on stiff substrates increased the migration speed and invasion potential in a similar way in 2D and 3D matrices.^71^ However, in chondrocytes, 3D culture was found to better maintain differentiated phenotype compared to 2D TCPS,^89^ the combined effect of low stiffness and 3D environment helping to limit any mechanical memory effect. To further confirm the occurrence of mechanical memory in 3D, more experiments are required in which mechanical stress or substrate stiffness are modulated after cell or organoid encapsulation. Tunable biomaterials allow in situ softening after encapsulation,^125,126^ and many more novel biomaterials with dynamic control of mechanical properties have recently been developed.^127,128^ In addition, there are also various methods to either directly apply mechanical stresses (atomic force microscopy, cell stretching) or to alter cell contractility that can be adapted to 3D systems.^78^ These toolboxes for control of cell and substrate mechanical properties will be useful in determining if cells in 3D culture are similarly sensitiveto mechanical memory as they are in 2D.

Finally, understanding how mechanical memory interplays with other factors, such as inflammation, could prove significant not only for in vitro bioengineering applications but also for treatment of pathologies, such as fibrotic diseases or cancer, in which the stiffness of the microenvironment is a key parameter: memory-erasing or mechanical reprogramming approaches could become therapeutically relevant in these contexts.
